# Analysis of Mobile App-Based Mental Health Solutions for College Students: A Rapid Review

**DOI:** 10.3390/healthcare11020272

**Published:** 2023-01-16

**Authors:** Avishek Choudhury, Annabella Kuehn, Hamid Shamszare, Yeganeh Shahsavar

**Affiliations:** Department of Industrial and Management Systems Engineering, Benjamin M. Statler College of Engineering and Mineral Resources, West Virginia University, Morgantown, WV 26506, USA

**Keywords:** digital health, young adults, depression, psychological distress, anxiety, stress

## Abstract

Background: College students are one of the most susceptible age groups to mental health problems. With the growing popularity of mobile health (mHealth), there is an increasing need to investigate its implications for mental health solutions. This review evaluates mHealth interventions for addressing mental health problems among college students. Methods: An online database search was conducted. Articles were required to focus on the impact of mHealth intervention on student mental health. Fifteen of the 487 articles, initially pulled from the search query, were included in the review. Results: The review identified three primary aspects of mental health: depression, anxiety, and stress. Research that found statistically significant improvements following mHealth intervention involved study durations between four and eight weeks, daily app use, guided lessons using cognitive behavioral therapy, acceptance and commitment therapy, and meditation. The review’s findings show that future work must address the concern of digital divide, gender and sex differences, and have larger sample sizes. Conclusions: There is potential to improve depressive symptoms and other similar mental health problems among college students via mobile app interventions. However, actions must be taken to improve barriers to communication and better reach the younger generations.

## 1. Introduction

Mental health can be defined as one’s condition regarding psychological, emotional, and social well-being. Instability of the mind can impact one’s thoughts, feelings, and actions [1]; thus, resulting challenges are expected. One of the most common mental health conditions is depression. The illness is recognized by a consistently low mood, loss of interest, and other debilitating emotions. Approximately 3.8% of the world is affected by depression. It is estimated that 20% of children and adolescents worldwide have some mental health problem, making it a global health concern [2].

Adolescents and young adults are a high risk for depression. According to the United States 2019 National Health Interview Survey, the highest rates of adult de-pression occurred among 18 to 29 year old (21% of participants had depressive symptoms) [2]. It is also reported that one in every five individuals experienced an episode by age 25 [3]. Within this age range falls a particular group of interest: college students. College students are known to be subject to high-stress environments, new experiences, and changing expectations during their education. In turn, they are particularly vulnerable to mental health challenges. It is estimated that one in every three college freshmen has mental health issues [4], and an estimated 9% of all students have depressive symptoms [5]. Unfortunately, public perception of mental illness is still recovering from barriers and past stigmatization. Whether or not people seek help, standard treatments are not always accessible; 95.6% of U.S. adults reported a barrier to mental healthcare access (the most critical link being affordability) [6]. Those affected often fail to recognize the severity of their conditions or fear being judged. College students are not immune to these barriers, typically being too embarrassed to seek treatment or wanting to work through their issues [7]. These beliefs can limit populations seeking traditional mental health treatments and pose challenges to awareness.

Mobile health (mHealth) technology has been growing in popularity to minimize some barriers to traditional mental healthcare [8,9]. The technology is unique for utilizing the increasing accessibility of mobile devices in the form of diagnostic apps, remote monitoring apps, mindfulness apps, text and video communication apps, and more. The technology has dramatically improved access to resources and treatment options across many populations. Young people and students are more likely to own and utilize mHealth apps. It is now timely and essential to evaluate the impact of mHealth treatments on students’ mental health.

In this study, we review the current research and successful techniques regarding mobile app mental health solutions for college students. The study will focus on depression and the ability of mobile apps to identify depressed students, present effective treatments for mental illness, and successfully rehabilitate users.

## 2. Materials and Methods

We leveraged the rapid review approach and analyzed information retrieved from PubMed and IEEE Xplore databases. Rapid reviews efficiently inform specific clinical or policy decisions promptly without losing much important information that may be expected from a comprehensive review. However, rapid reviews, although not exhaustive, should not be viewed as inherently inferior to full systematic reviews [10]. This approach is best suited for capturing timely information about fast-growing topics [8]. Synthesizing a standard systematic review, which is best suited to assess well-established topics, typically takes significant time to evaluate and analyze at least ten years of literature; therefore, fails to capture emerging issues. A rapid review speeds up the systematic review process by omitting stages of the systematic review making it less rigorous and precise [8,9].

### 2.1. Search Query

The keywords used to search for the papers were “students”, “mental health”, and “mobile” or “app”. The query used for students was: “student’s” [All Fields] OR “students” [MeSH Terms] OR “students” [All Fields] OR “student” [All Fields] OR “students” [All Fields]. The query used for mental health was: “mental health” [MeSH Terms] OR (“mental” [All Fields] AND “health” [All Fields]) OR “mental health” [All Fields]. The query used for mobile was: “mobile” [All Fields] OR “mobiles” [All Fields] OR “app” [All Fields].

### 2.2. Selection Criteria

Studies that tested for mental health problems related to depression were considered for evaluation. We considered studies that implemented a mobile app to improve depressive symptoms in young adults. Articles had to have focused on improving students’ mental health or the young adult populations using mHealth intervention. Note that the articles had to analyze the intervention’s impact, not just its feasibility. Articles older than five years were also removed from evaluation. The review included only articles published in English; the country of origin was not restricted. Apart from the desired information, the search resulted in many articles regarding mobile phone addiction and related issues, which were removed from the review.

Abstracts and, eventually, full articles were reviewed for their acceptability of the nature of this review. Two graduate students independently reviewed and coded the papers achieving an inter-reliability of 0.83. A third senior researcher then resolved the conflicts.

### 2.3. Coding and Data Extraction

Selected articles were categorized by country of origin, name of the app used, app’s accessibility (whether the app was accessible to the public or only to study participants), app methodology (the method of impacting the mental health of the users), intervention method (the mode via which the apps communicated with users), frequency of app use, duration of intervention, number of participants, participant dropout rate, participant gender, and ethnicity. We also captured the different mental health aspects analyzed by the studies and identified successful interventions where significant improvements were made.

## 3. Results

**Figure 1:**
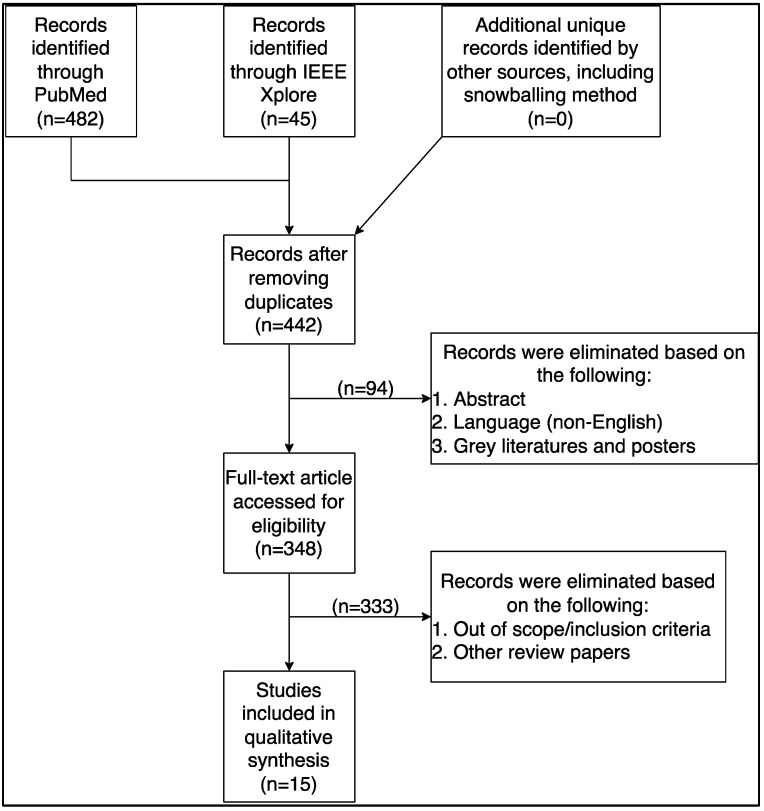
The rapid review selection process.

The search query yielded 487 (PubMed = 442, IEEE Xplore = 54) articles, of which articles published in the last five years were kept. Older articles or articles published in foreign languages were also excluded (*n* = 94). Review articles, conference abstracts, posters, opinion papers, letters to editors, editorials, book chapters, and other grey literature were excluded (*n* = 27). Others (*n* = 368) did not match our inclusion criteria and were excluded. Fifteen articles matched the inclusion criteria and were analyzed in this study. Figure 1 illustrates the review process.

As shown in Table 1, these studies were performed in developed nations across seven different countries, of which one was a multinational study [11], and one did not report their location [12]. Most studies were done in the United States of America [13,14,15,16,17] (*n* = 5), followed by Germany [18,19,20] (*n* = 3). The following countries are based on only one study each: Canada [21], Japan [22], Scotland [23], South Korea [24], and Sweden [25]. As shown in Table 1, all studies had significantly more female participants than male or other genders. On average, 72% of subjects identified as female in the evaluated studies. Half of the studies (50%) published the racial demographics of their subjects, of which 47% of the studies had minority representation in their participant pool. The studies had varying dropout rates that could be based on several factors. One study maintained all participants from baseline to completion [14]. Conversely, in another study, dropout rates reached 34% [23]. The studies averaged a 13% reduction from the baseline participant population to the final remarks.

### 3.1. Mobile App for Mental Health

As shown in Table 2, thirteen different apps were used across the fifteen studies. Most of the apps evaluated were available for public use before the study. [13,14,15,17,18,19,21,23] The other apps were created for their respective studies and await validation before seeking more widespread audiences [11,12,16,20,22,24,25]. The Calm app [15,23] and the StudiCare Stress app [11,20] were used by two studies, respectively. Other apps used by different studies included the ACT Daily app, [14] Balloon app, [19] DeStressify app [21], IntelliCare for College Students app [16], K-CESD-R Mobile App app [24], MCT & More app [18], Mental app [22], Metric Wire app [12], mHealth Positive Psychology Multicomponent Program app [25], Nod app [13], and Stop, Breathe and Think app [17]. Most apps leveraged a combination of methodologies to deliver diagnosis and treatment. The most common strategies were meditation and mindfulness [12,13,15,17,18,19,20,21,23] (*n* = 8), cognitive behavioral therapy [12,13,15,18,20,23] (*n* = 5), acceptance and commitment therapy [14,18,20] (*n* = 3), metacognitive training [18,19] (*n* = 2), and positive psychology [13,25] (*n* = 2). Other strategies enacted included varying kinds of surveys and emotion regulation strategies. The apps delivered these strategies via different means of presentations. Most apps (*n* = 9) used traditional guided exercises and lessons to impart their knowledge to users [11,14,15,16,17,18,19,20,21,23,25]. Other apps used combinations of virtual workshops, reflections, and self-monitoring [12,13,22,24]. The StudiCare Stress app and ACT Daily app used chatbots to interact with users and guide them through lessons. All apps used questionnaires and surveys to gauge user feelings.

Participants of the different studies were asked to use their respective apps for varying degrees of time. The most common assignment revolved around using the app once daily. This could be one prompt, one session, or one exercise daily, usually taking about ten minutes to complete [15,16,17,19,23,24,25]. Few studies required the participants to use the app once or twice every week [11,20]. Other studies required participants to use the app multiple times a day [12,14]; Some studies also left the frequency of interaction entirely up to users [13,22]. The most common intervention length was four weeks [13,17,18,21] and eight weeks [12,15,16,19]. The most extended intervention lasted ten weeks, [25] while the shortest lasted one week [23]. Post-evaluation follow-ups were noted in eight studies [11,13,14,15,20,21,23,25].

### 3.2. Mental Health Attributes and Assessment

Table 3 shows the three primary and five secondary attributes of mental ailments evaluated across different studies (primary: depression, anxiety, and stress; secondary: sleep quality, self-image, burnout, perception of quality of life, and general health). These attributes were selected in this review because of their relevance to depressive symptoms and frequency of use. The mental health attributes (themes) were noted at face value and reflected the studies’ definitions.

Twelve studies in this review tested the effectiveness of their app on the depression [11,12,13,14,16,17,18,20,21,22,24,25]. Six of those twelve studies found significant improvements in their participants’ symptoms before and after the intervention [11,12,14,18,20,25]. Two studies found no effective results but indications of substantial potential developments in the future [16,24]. Ten tested the effectiveness of their app in improving anxiety [11,12,13,14,16,17,20,21,24,25]. Of those ten studies, seven found significant improvements in their participants’ symptoms from before to after intervention [11,12,14,17,20,21,25]. Seven in this review tested the effectiveness of their app on stress [11,14,15,16,19,20,21]. Of those seven studies, six found significant improvements in their participants’ symptoms from before to after intervention [11,14,15,16,19,20]. Three studies tested the effectiveness of their app on sleep quality [13,15,21]. Of those three studies, one found significant improvements in their participants’ symptoms from before to after intervention [15]. Four studies tested the effectiveness of their app on self-image [11,15,18,20], of which three found significant improvements in their participants’ symptoms from before to after intervention [11,15,18]. Two studies tested the effectiveness of their app on burnout and found significant improvements in their participants’ symptoms from before to after intervention [11,20]. Three studies tested their app’s effectiveness on the perception of quality of life [18,19,21], of which one found significant improvements in their participants’ symptoms from before to after intervention [21]. Five studies tested the effectiveness of their app on general health and found significant improvements in their participants’ symptoms from before to after intervention [17,20,22,23,25].

Table 4 summarizes all the validated scales used by different studies to evaluate various aspects of mental health. Stress was measured using (a) Presenteeism Scale for Students (PSS) and (b) Depression, Anxiety, and Stress Scale (DASS). Depression was measured using the following eight different validated scales: (a) Center for Epidemiological Studies Depression Scale (CES-D), (b) Hospital Anxiety Depression Scale (HADS), (c) Patient Health Questionnaire (PHQ), (d) Quick Inventory for Depressive Symptomatology Self Report (QIDS-SR), (e) Montgomery-Asberg Depression Rating Scale (MADRS), (f) Hamilton Depression Rating Scale (HAM-D), (g) Ecological Momentary Assessment (EMA), and (h) DASS. Anxiety was measured using the following seven validated scales: (a) Spielberger State-Train Anxiety Inventory, (b) HADS, (c) Generalized Anxiety Disorder Scale (GAD), (d) Mini Social Phobia Inventory (MSPI), (e) Hamilton Anxiety Rating Scale (HAM-A), (f) EMA, and (g) DASS.

## 4. Discussion

### 4.1. Main Findings

Our review indicates a lack of studies evaluating the effectiveness of mHealth on students’ mental health problems and identified only fifteen published research. Since mHealth is rapidly increasing in popularity and the feasibility of such interventions is well accepted, the amount of data proving the best implementation method is underwhelming. There is a significant gap in overall research on the validity of mental health-based mobile apps.

When aiming to improve depression, cognitive behavioral therapy, acceptance and commitment therapy, mindfulness, positive psychology, and emotion regulation strategies were effective. Our review indicates that self-reflection is generally ineffective in addressing anxiety issues, meaning anxious participants may prefer to follow lessons than revisit past trauma. We also noted that longer intervention times (at least seven weeks) were most effective, meaning a more prolonged exposure to the app may help improve mental health conditions. Consistent and regular mindfulness practices via the mHealth app effectively improved the sleep quality of study participants across multiple studies. In improving the perception of self-image, guided lessons via mHealth apps were effective. Looking at burnout, improvements most often result in more extended intervention periods. The impact of mHealth on students’ general health perception was successful, where all five studies analyzed for the factor noted significant improvements in participants.

Our review noted a wide variety of interventions design across different studies, implying a need for further research to standardize the approach for optimal outcomes. In our review, the ideal setting for successfully improving the mental health of college students via mobile apps includes the following: one daily prompt averaging about 10 min; delivery via traditional guided exercises and lessons; content based on cognitive behavior therapy, acceptance and commitment therapy, and meditation; interventions lasting four to eight weeks with a follow-up.

### 4.2. Digital Divide

All fifteen studies identified in this review were conducted in high-income or developed nations, indicating the need for evaluating mHealth’s effectiveness on underserved and vulnerable populations across low-and-middle-income countries (LMICs). Compared to most other mobile technology, smartphones have penetrated and reached many rural and underserved regions across the globe, augmenting the potential of mHealth to improve access to affordable healthcare [26,27]. For many in LMICs, smartphones are often the only way to connect to the internet and access information and services, including healthcare. Unfortunately, mHealth and other mobile-based apps, instead of bridging the digital divide in healthcare, are contributing to the problem [28]. The digital divide results in unequal access to digital technology, resulting in inequality around access to resources, including healthcare services.

It must be understood that digital interventions must be user-centered, i.e., the technology interface, usability, and intervention design should cater to the end users’ needs. That being said, a mobile app designed and evaluated on a particular stratum of the population in a developed nation will not yield similar outcomes if implemented in underserved societies in a low-income country. Therefore, future studies should evaluate mHealth apps on a more diverse user across LMICs.

### 4.3. Gender Disparity and Sample Size

Sex and gender differences are often overlooked in research design. Our review identified a skewed representation of both. Unlike most other studies where female underrepresentation has been an issue [29], our review identified the underrepresentation of males. This oversight hinders the generalizability of research findings and their applicability to clinical practice. Future research must work to close gaps in equal sex and gender representation.

We also noted heterogeneity in the sample across all studies. Future studies should prepare bigger sample sizes to obtain generalizable outcomes and compensate for high dropout rates. A dropout rate in any research is the percentage of participants who do not complete the research task due to the study duration, lack of interest, technical difficulties, poor design, or insufficient compensation. As noted in our review, studies have faced a dropout rate of up to 50%. Dropout in research typically diminishes the validity of results, as completers may differ from people who drop out [30]. It may also result in a biased sample. Therefore, standardization must be made to inform the optimal sample size and diversity required to obtain feasible research validity.

### 4.4. Recommendations

Several actions could be taken to improve the acceptance and effectiveness of mHealth interventions among students:

Integrate mHealth interventions and not impose: To ensure the sustainability and effectiveness of mHealth interventions, it is essential to integrate them into the current lifestyle of students (study participants) and to work with them (and family) to ensure that interventions are aligned with their priorities and needs. It is also essential to consider human factors while designing the study and catering the comprehensive technology to meet students’ needs. Using the Test of Attentional and Interpersonal Style questionnaire [31], researchers should determine the information architecture to match user preference.

Ensure that mHealth interventions are culturally and linguistically appropriate: To ensure the adoption and effectiveness of mHealth interventions, it is essential to ensure that interventions are culturally and linguistically appropriate. Although international students are fluent in English, having multiple language choices built into the app would drastically change how they perceive the technology. However, studies should ensure that different languages retain common meanings and semantics.

Conduct ongoing evaluation and monitoring of mHealth interventions: It is essential to conduct ongoing evaluation and monitoring to identify and address any challenges or issues that may arise. This could involve using various evaluation and monitoring tools, such as surveys, focus groups, and data analysis.

Implement strong privacy and security measures: To ensure the privacy and confidentiality of personal and health information, it is essential to implement strong privacy and security measures for mHealth interventions [32]. This could involve implementing secure data storage and transmission systems and developing policies and procedures to protect personal and health information. More importantly, the student must be aware of the strengths of the security system.

### 4.5. Limitations

This study encompasses publications that matched our inclusion criteria. Therefore, the review is limited to relevant studies published in English between January 2017 and September 2022.

## 5. Conclusions

With the introduction and growing popularity of mHealth solutions, there has been a rise in the use of mobile apps to combat mental health problems. Our review shows mHealth’s potential to improve depressive symptoms and other similar mental health problems among college students. However, actions must be taken to improve barriers to communication and better reach the younger generations in healthcare. Besides, concerns such as digital divide, and unequal sex and gender representation, can hinder the growth, generalizability, effectiveness, and overall acceptance of mHealth for mental health.

## Figures and Tables

**Table 1:** Study and participant characteristics.

Location	Intervention	Follow-Up	Participants at Baseline	Dropout Rate	Female	Minority Race
	Weeks	Weeks	*n*	%	*n* (%)	%
[15] USA	8	4	88	0	79 (88)	39
[20] Multinational	7	5	150	30	112 (75)	Na
[25] Sweden	10	2	654	48	510 (78)	Na
[16] USA	8	None	21	1	17 (81)	28
[21] Canada	4	4	206	21	103 (63)	21
[18] Germany	4	None	400	34	357 (89)	Na
[19] Germany	8	None	99	35	67 (68)	Na
[22] Japan	2	None	68	16	17 (30)	Na
[13] USA	4	4	221	5	131 (59)	47
[11] Germany	7	5	200	24	170 (85)	Na
[24] South Korea	2	None	70	7	33 (51)	Na
[23] Scotland	1	2	269	50	218 (81)	16
[17] USA	4	None	23	30	16 (100)	0
[12] Unidentified	8	None	222	20	138 (78)	67
[14] USA	2	2	11	0	9 (82)	9

Na: not available.

**Table 2:** mHealth apps used by different studies.

Name of the App	Access to the App	Methodology of the App	Intervention Delivery Approach	Frequency of App Use
[15] Calm	Public	Meditation, CBT	TGE	10 min Daily
[20] StudiCare Stress	Participants	CBT and Mindfulness, Emotions, Acceptance	TGE, e-Coach (chatbot)	Two weekly modules
[25] mHealth PPMP	Participants	Positive Psychology	Text Messages, TGE	10 min Daily
[16] IntelliCare for College Students	Participants	Cognitive Restructuring, Behavioral Activation	Mood Rating and Journal, Symptom Check, Lessons, and Resources	1 Text daily
[21] DeStressify	Public	Mindfulness	TGE	5 days/week)
[18] MCT & More	Public	CBT, Mindfulness, ACT, Metacognitive Training	TGE	3–10 min daily
[19] Balloon	Public	MCT, Mindfulness, and Meditation	TGE	10 min daily
[22] Mental App	Participants	Focus on Diagnosis Rather Than Treatment	Self-Monitoring, Self-Screening, and Referrals	Not defined
[13] Nod	Public	Positive Psychology, Mindfulness, CBT	Social Challenges, Reflections, and Written Testimonials	Not defined
[11] StudiCare	Participants	ERS	TGE	1 weekly module
[24] K-CESD-R Mobile App	Participants	K-CESD-R Surveys	K-CESD-R Surveys	1 session daily
[23] Calm	Public	Meditation, CBT	TGE	10 min daily
[17] Stop, Breathe, and Think	Public	Meditation	TGE, Meditation and Emotional Check-Ins	1 prompt daily
[12] MetricWire	Study	Mindfulness, Cognitive Flexibility, ERS	Therapeutic Workshop Skills	4 prompts daily
[14] ACT Daily	Public	ACT	Coaching Sessions	3 questionnaires daily

ACT: Acceptance and Commitment Therapy; CBT: Cognitive Behavioral Therapy. TGE: Tradi-tional Guided Exercises; PPMP: Positive Psychology Multicomponent Program. ERS: Emotional Regulation Strategies.

**Table 3:** Study outcomes.

	Mental Health Factors (Primary)			Other Factors Related to Mental Health (Secondary)				
Study	Depression	Anxiety	Stress	Sleep Quality	Self-Image	Burnout	Quality of Life	General Health
[15]			Y *	Y *	Y *			
[20]	Y *	Y *	Y *		Y	Y *		Y *
[25]	Y *	Y *						Y *
[16]	Y	Y	Y *					
[21]	Y	Y *	Y	Y			Y *	
[18]	Y *				Y *		Y	
[19]			Y *				Y	
[22]	Y							Y *
[13]	Y	Y		Y				
[11]	Y *	Y *	Y *		Y *	Y *		
[24]	Y	Y						
[23]								Y
[17]	Y	Y *						Y *
[12]	Y *	Y *						
[14]	Y *	Y *	Y *					

* Significant improvement after mHealth intervention. Y: factors measured by different studies.

**Table 4:** Validated scales used in different studies to evaluate various attributes of mental health.

Study	Validated Scales Used by Different Studies to Measure Different Mental Health Factor
	Mental Health and Related Factor (Scales Used)
[15]	Stress (PSS), Mindfulness (FFMQ), Self-Compassion (SCS-SF), Sleep Quality (PROMIS), Binge Drinking (YRBS), Physical Activity Participation (YRBS), Healthy Eating (YRBS)
[20]	Stress (PSS), Depression (CES-D), Anxiety (STAI), General Well-Being (WHO-5), Emotional Exhaustion (MBI-S), Dysfunctional Perfectionism (RAPS), Resilience (CD-RISC), Self-Compassion (SCS), Self-Esteem (RSES), Work Impairment (WIS), Academic Self-Efficacy (ASES), Academic Worrying (AWQ)
[25]	Mental Health and Well Being (MHC-SF), Depression (HADS), Anxiety (HADS)
[16]	Depression (PHQ), Anxiety (GAD), Anxiety Literacy (ALQ), Depression Literacy (DLQ), Knowledge and Beliefs About Services (KBSS), Cognitive and Behavioral Response to Stress (CB-RSS)
[21]	Stress (PSS), Anxiety (STAI), Depression (QIDS-SR), Sleep Quality (PSQI), Quality of Life (RAND-HS), Work Productivity (WPAI)
[18]	Depression (PHQ), Self-Esteem (RSES), Quality of Life (WHOQOL-BREF), Attitude Towards Psychological Online Interventions (APOI), Patient Therapy Expectation and Evaluation (PATHEV), Negative Effects of Psychotherapy (INEP)
[19]	Stress (PSS), Self-Regulation (SRS), Life Satisfaction and Happiness (QAH), Mindfulness (FMI), Emotional Regulation (ERQ), Social Desirability (SEA)
[22]	Public Stigma (LSS), Depression (CES-D), General Health (GHQ)
[13]	Loneliness (UCLA), Anxiety (GAD), Depression (PHQ), Social Anxiety (MSPI), Sleep Quality (PSQI), Perceived Social Support (CIT), Campus Belonging (SERUQ), Social Adjustment to College (SACQ), Intention to Return to College (NSSE)
[11]	Depression (CES-D), Behavioral Activation for Depression (BADS), Stress (PSS), Anxiety (STAD), Worrying (AWQ), Emotional Exhaustion (MBI-S), Work Impairment (WIS), Work Output (WOS), Work Cutback (PS-S), College Self-Efficacy (CSED), Resilience (CD-RISC), Emotion Regulation Competencies (SEK), Self-Compassion (SCS-D), Self-Esteem (RSES), Negative Beliefs About Stress (BASS), Positive Beliefs About Stress (BASS), Controllability Beliefs About Stress (BASS)
[24]	Depression (PHQ, CES-D, QIDS-SR, MADRS, HAM-D), Anxiety (HAM-A), Severity of Illness (CGI-S), Neuropsychiatric Interview (MINI)
[23]	Mindfulness (FFMQ), Generalized Self-Efficacy (GSE), Mental Well-Being (SWEMWBS)
[17]	Positive Mental Health (CCAPS, MHC-SF), Mindfulness (FFMQ), Values Progress (V.Q.)
[12]	Depression (EMA), Anxiety (EMA)
[14]	Depression (DASS), Anxiety (DASS), Stress (DASS), Psychological Inflexibility (AAQ), Cognitive Fusion (CFQ), Mindfulness (PHLMS), Valued Living (V.Q.), Emotional Self-Awareness (ESAS)

AAQ: Acceptance and Action Questionnaire; ALQ: Anxiety Literacy Questionnaire; APOL: Attitude Towards Psychological Online Interventions; ASES: Academic Self-Efficacy Scale; AWO: Academic Worrying Questionnaire; BADS: Behavioral Activation for Depression Scale; BASS: Beliefs About Stress Scale; CB-RSS: Cognitive and Behavioral Response to Stress Scale; CCAPS: Counseling Center Assessment of Psychological Symptoms; CD-RISC: Connor-Davidson Resilience Scale Short Form; CES-D: Center for Epidemiological Studies’ Depression Scale; CPQ: Cognitive Fusion Questionnaire; CGI-S: Clinical Global Impressions Severity of Illness Scale; CIT: Comprehensive Inventory for Thriving; CSEI: College Self-Efficacy Inventory; DASS: Depression, Anxiety, and Stress Scale; DLQ: Depression Literacy Questionnaire; EMA-SI: Ecological Momentary Assessment Suicidal Ideation Questions; EMA: Ecological Momentary Assessment; ERQ: Emotion Regulation Questionnaire; ESAS: Emotional Self-Awareness Scale; FFMQ: Five Factor Mindfulness Questionnaire; FMI: Freiburg Mindfulness Inventory; GAD: Generalized Anxiety Disorder Scale; GHQ: General Health Questionnaire; GSE: Generalized Self Efficacy Scale; HADS: Hospital Anxiety Depression Scale; HAM-A: Hamilton Anxiety Rating Scale; HAM-D: Hamilton Depression Rating Scale; INEP: Inventory for Assessing Negative Effects of Psychotherapy; INQ: Interpersonal Needs Questionnaire; KBSS: Knowledge and Beliefs about Services Scale; LSS: Link Stigma Scale; MADRS; Montgomery-Asberg Depression Rating Scale; MBI-S: Maslach Burnout Inventory Student Version; MHC-SF: Mental Health Continuum Short Form; MINI: Mini-Intonational Neuropsychiatric Interview; MSPL: Mini Social Phobia Inventory; NSSE: National Survey for Student Engagement; PANAS: Positive and Negative Affect Schedule Scale; PATHEV: Patient Questionnaire on Therapy Expectation and Evaluation; PHLMS: Philadelphia Mindfulness Scale; PHQ: Patient Health Questionnaire; PIL: Purpose in Life Seale; PROMIS: Patient-Reported Outcomes Measurement Information System; PS-S: Presenteeism Scale for Students; PSQI: Pittsburg Sleep Quality Index; PSS: Perceived Stress Scale; QAH: Questionnaire for the Assessment of Happiness; QIDS-SR: Quick Inventory of Depressive Symptomatology Self-Report; RAND-HS: RAND Health Survey; RAPS: Revised Almost Perfect Scale; RSES: Rosenberg Self-Esteem Scale; SACQ: Student Adaptation to College Questionnaire; SCS-D: Self-Compassion Scale; SCS-SF: Self-Compassion Scale Short Form; SEA: Short Form Scale to Detect False Self-Representation; SEK: Assessment of Emotional Regulation Skills; SERUQ: Student Experiences in the Research University Questionnaire; SRS: Self-Regulation Scale; SWEMWBS: Short Warwick-Edinburgh Mental Well-Being Scale; UCLA: UCLA Loneliness Questionnaire; V.Q.: Valuing Questionnaire; WHO-5: WHO Well-Being Index; WHOQOL-BREF: World Health Organization Quality of Life Abbreviated Version; WIS: Work Impairment Scale; WOS: Work Output Scale; WPAL: Work Productivity and Activity Impairment Questionnaire; YRBS: Youth Risk Behavior Surveillance Survey.

## Data Availability

Not applicable.
